# Use of implementation mapping to develop a multifaceted implementation strategy for an electronic prospective surveillance model for cancer rehabilitation

**DOI:** 10.1186/s43058-024-00650-4

**Published:** 2024-10-01

**Authors:** Christian J. Lopez, Sarah E. Neil-Sztramko, Mounir Tanyoas, Kristin L. Campbell, Jackie L. Bender, Gillian Strudwick, David M. Langelier, Tony Reiman, Jonathan Greenland, Jennifer M. Jones, on behalf of the Canadian Cancer Rehabilitation Team

**Affiliations:** 1 Department of Supportive Care, Princess Margaret Cancer Centre, Toronto, Ontario Canada; 2 Institute of Medical Science, University of Toronto, Toronto, Ontario Canada; 3 Faculty of Health Sciences, McMaster University, Hamilton, Ontario Canada; 4 National Collaborating Centre for Methods and Tools, McMaster University, Hamilton, Ontario Canada; 5 Department of Physical Therapy, University of British Columbia, Vancouver, British Columbia Canada; 6 Dalla Lana School of Public Health, University of Toronto, Toronto, Ontario Canada; 7 Institute of Health Policy, Management and Evaluation, University of Toronto, Toronto, Ontario Canada; 8 Department of Clinical Neurosciences, Division of PM&R, University of Calgary, Calgary, Alberta Canada; 9 Department of Oncology, Saint John Regional Hospital, Saint John, New Brunswick Canada; 10 Department of Biological Sciences, University of New Brunswick, Saint John, New Brunswick Canada; 11 Faculty of Medicine, Memorial University of Newfoundland, St John’s, Newfoundland Canada; 12 Dr. H. Bliss Murphy Cancer Centre, Eastern Health, St. John’s, Newfoundland Canada

**Keywords:** Implementation science, Implementation mapping, Consolidated framework for implementation research, Knowledge to action framework, Expert recommendations for implementing change, Implementation strategies, Cancer survivorship, Prospective surveillance model, Patient-reported outcomes, Rehabilitation

## Abstract

**Background:**

Electronic Prospective Surveillance Models (ePSMs) remotely monitor the rehabilitation needs of people with cancer via patient-reported outcomes at pre-defined time points during cancer care and deliver support, including links to self-management education and community programs, and recommendations for further clinical screening and rehabilitation referrals. Previous guidance on implementing ePSMs lacks sufficient detail on approaches to select implementation strategies for these systems. The purpose of this article is to describe how we developed an implementation plan for REACH, an ePSM system designed for breast, colorectal, lymphoma, and head and neck cancers.

**Methods:**

Implementation Mapping guided the process of developing the implementation plan. We integrated findings from a scoping review and qualitative study our team conducted to identify determinants to implementation, implementation actors and actions, and relevant outcomes. Determinants were categorized using the Consolidated Framework for Implementation Research (CFIR), and the implementation outcomes taxonomy guided the identification of outcomes. Next, determinants were mapped to the Expert Recommendations for Implementing Change (ERIC) taxonomy of strategies using the CFIR-ERIC Matching Tool. The list of strategies produced was refined through discussion amongst our team and feedback from knowledge users considering each strategy’s feasibility and importance rating via the Go-Zone plot, feasibility and applicability to the clinical contexts, and use among other ePSMs reported in our scoping review.

**Results:**

Of the 39 CFIR constructs, 22 were identified as relevant determinants. Clinic managers, information technology teams, and healthcare providers with key roles in patient education were identified as important actors. The CFIR-ERIC Matching Tool resulted in 50 strategies with Level 1 endorsement and 13 strategies with Level 2 endorsement. The final list of strategies included 1) purposefully re-examine the implementation, 2) tailor strategies, 3) change record systems, 4) conduct educational meetings, 5) distribute educational materials, 6) intervene with patients to enhance uptake and adherence, 7) centralize technical assistance, and 8) use advisory boards and workgroups.

**Conclusion:**

We present a generalizable method that incorporates steps from Implementation Mapping, engages various knowledge users, and leverages implementation science frameworks to facilitate the development of an implementation strategy. An evaluation of implementation success using the implementation outcomes framework is underway.

**Supplementary Information:**

The online version contains supplementary material available at 10.1186/s43058-024-00650-4.

Contributions to the Literature

• Previous guidance on implementing electronic Prospective Surveillance Models (ePSMs) in cancer care lacks sufficient detail on approaches to select implementation strategies for these systems.
• We present a case example of selecting strategies for an ePSM using Implementation Mapping methodology that involved meaningful stakeholder feedback and incorporated additional implementation science frameworks, including the Consolidated Framework for Implementation Research and the Expert Recommendations for Implementing Change taxonomy.
• The methodology described in this article can provide meaningful guidance for decision-makers and implementers interested in integrating this cost-effective model into their practice to facilitate the early identification and management of cancer-related impairments.

## Background

Cancer-related impairments are commonly experienced during and after treatment and are a major cause of disability [1–3]. However, despite clinical practice guidelines defining rehabilitation as an essential component of survivorship care [4], there is a significant mismatch between the high incidence of cancer-related impairments, the timely detection of rehabilitation needs, and the utilization of rehabilitation services [5–8]. As such, novel approaches to optimize the identification of rehabilitation needs along the cancer care continuum are needed.

Digital solutions present an opportunity to address unmet needs in managing cancer-related impairments [9]. For instance, a Prospective Surveillance Model (PSM) for cancer rehabilitation has been identified as an effective solution to identify and meet the needs of people with cancer [10, 11]. A PSM involves standardized assessments of rehabilitation needs during cancer care to facilitate the identification and management of cancer-related impairments [12]. While originally developed for ongoing surveillance efforts with repeated clinic-based assessments by an interdisciplinary team of providers, this model can be delivered electronically (an electronic PSM (ePSM)) to address common organizational and resource barriers including staff and infrastructure needed to conduct these assessments [10, 11].

**Fig. 1:**
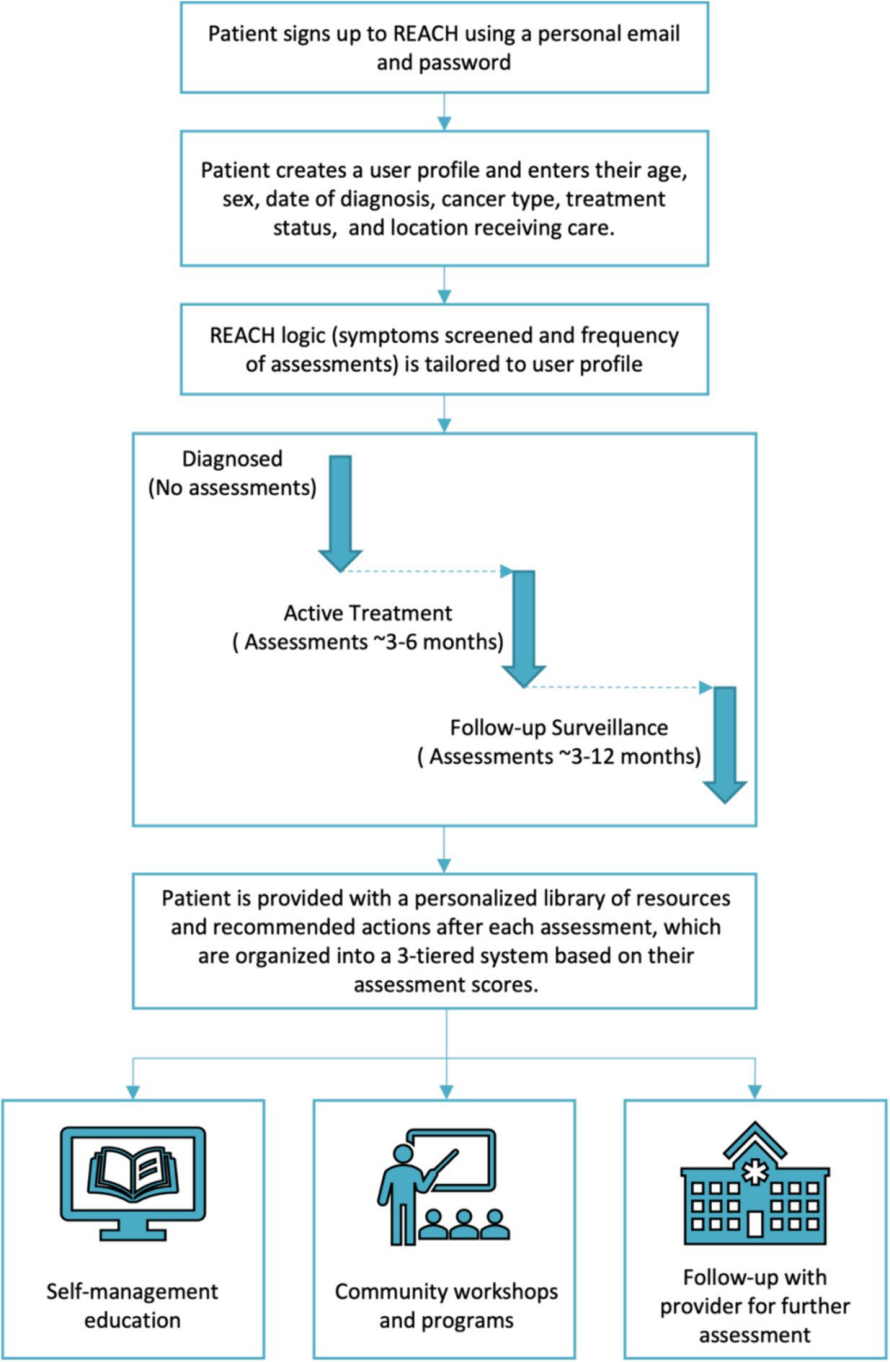
Description of the use of the REACH system

An ePSM uses electronic patient-reported outcomes (ePROs) to monitor symptoms during cancer care and subsequently provides links to self-management education or alerts for additional screening by the clinical team and referrals to rehabilitation services. Randomized controlled trials have demonstrated that these electronic tools can effectively identify cancer-related impairments early, resulting in better management [13–15], which supports the need to implement these systems into routine clinical practice. As such, our team developed REACH, a web-based application that can be accessed by patients using any electronic device and is designed to monitor physical cancer-related impairments for four cancer types (breast, colorectal, lymphoma, and head and neck) from the start of treatment to two years post-treatment. All patients on REACH are assessed for fatigue, pain, activities of daily living, and falls and balance, with additional impairments assessed for each cancer type such as dysphagia and trismus (head and neck), lymphedema (breast), and sexual function (breast and colorectal). Based on the reported level of impairment, REACH provides patients with recommended educational resources for self-management and/or rehabilitation programs (Fig. 1).

While there is substantial evidence supporting the effectiveness of ePSMs, there is a limited understanding regarding the optimal methods for integrating them into the delivery of cancer care [16]. Implementation science provides an evidence-based and theory-informed approach to guide the implementation of ePSMs in cancer care, including identifying key factors that may influence implementation and selecting effective implementation strategies to address these factors (i.e., methods or techniques used to enhance implementation) [17]. Mapping barriers and facilitators to specific implementation strategies has been suggested to mitigate barriers [18] and tailoring implementation strategies to a given context can increase implementation success [19, 20]. However, selecting the most appropriate implementation strategies is a complex process that requires the consideration of theory, the type of evidence-based intervention being implemented, and the context and characteristics of the clinical setting [21]. While several published reports have recommended steps to implement tools that use ePROs in cancer [22–24], including suggestions for how to assess the clinical context and select tailored strategies using implementation science frameworks [25], there are limited examples of how to select implementation strategies in real-world implementation efforts of ePSMs. This article aims to describe the development of a multifaceted implementation strategy for the REACH ePSM system.

## Methods

### Theoretical frameworks and preliminary work

**Fig. 2:**
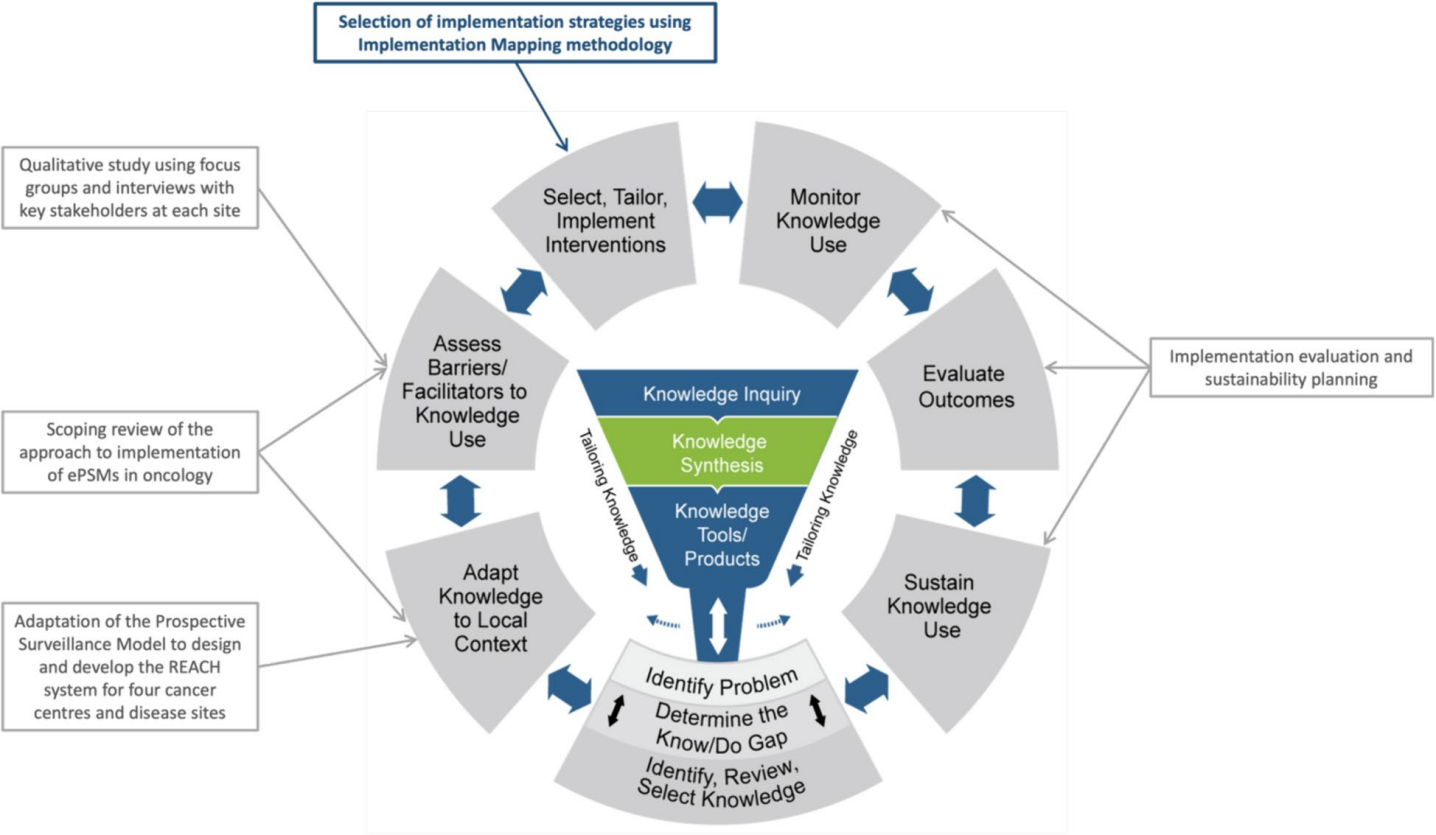
Use of the Knowledge-to-Action Cycle for the implementation of REACH. Adapted from Graham ID, Logan J, Harrison MB, Straus SE, Tetroe J, Caswell W, et al. Lost in knowledge translation: time for a map? J Contin Educ Health Prof. 2006;26(1):13-24

This work is part of a multi-phase implementation study including the development and design of the REACH system, the development of the implementation plan, the integration of REACH into routine clinical care across four regional cancer centres in Canada, and the evaluation of its implementation and sustainability. The Knowledge-to-Action (KTA) cycle [26] was used as the guiding process model to inform the entire REACH initiative, encompassing all phases from its development to its implementation (Fig. 2). Across each of KTA phases, we have used a variety of methods, models, and frameworks tailored to the objectives of each phase. For instance, as part of the KTA phases 'adapt knowledge to local context', and 'assess barriers and facilitators to knowledge use', we conducted a scoping review on the approach to implementing ePSM interventions in cancer care and pre-implementation qualitative interviews with various knowledge users (i.e., patients, healthcare providers (HCPs), and clinic management). Full details of the methods and results of both studies have been published [27, 28]. Importantly, both studies utilized the initial version of Consolidated Framework for Implementation Research (CFIR) [29] to categorize barriers and facilitators to implementation. Additionally, the scoping review used the Expert Recommendations for Implementing Change (ERIC) taxonomy [30, 31] to categorize the various strategies used to support the implementation of ePSMs, as well as the implementation outcomes taxonomy [32] to categorize outcomes used to evaluate the implementation of these systems. Further, the pre-implementation qualitative study captured potential implementation actions and actors to support the implementation of REACH. The implementation data collected from our scoping review and qualitative study provided the foundation for selecting implementation strategies that target various actors, actions and outcomes. This article describes how these results were synthesized and utilized to select the implementation strategies for REACH.

Following this preliminary work, we aimed to conduct the KTA phase ‘select and tailor implementation strategies’ (Fig. 1). To guide the application of this phase, we followed the steps outlined in Implementation Mapping (IM) [33]. IM provides a systematic five-step process for selecting implementation strategies, which include: 1) conduct an implementation needs assessment; 2) identify adoption and implementation outcomes, performance objectives, determinants, and change objectives; 3) select theoretical change methods and design implementation strategies; 4) produce implementation protocols and materials; and 5) evaluate implementation outcomes. This article describes the process we undertook in Steps 1–4 to develop implementation strategies for REACH, along with the various implementation science frameworks and tools employed within each step (Table 1). This process involved oversight and feedback from REACH project’s Steering Committee, Patient and Family Advisory Committee (PFAC), advisors with expertise in implementing digital health interventions within clinical care practices (JLB and GS), and HCPs at each of the four cancer centres where REACH is being implemented. The involvement of these various stakeholders is specified within each step described below. Steps 1 and 2 were completed iteratively and are reported together. A pragmatic evaluation of implementation is underway (Step 5). Reporting of this process followed the Standards for Reporting Implementation Studies (Additional file 1).

**Table 1:** Summary of implementation mapping steps conducted for REACH

KTA Step: Select and Tailor Implementation Strategies		
Implementation Mapping Steps	Guiding Implementation Frameworks/Tools	Objectives and Methods
1. Conduct implementation needs assessment	CFIR Implementation Outcomes Taxonomy	• Synthesize and categorize both lists of barriers and facilitators identified in our scoping review and pre-implementation qualitative study via the CFIR • Identify relevant implementation actors and actions using findings from our pre-implementation qualitative study • Identify relevant implementation outcomes and measures using findings from our scoping review
2. Identify adoption and implementation outcomes, performance objectives, determinants, and change objectives		
3. Select theoretical change methods and design implementation strategies	CFIR-ERIC Implementation Strategy Matching Tool Go-Zone plot	• Develop a preliminary list of implementation strategies by inputting the CFIR barriers identified in Step 1 into the CFIR-ERIC Implementation Strategy Matching Tool • For each strategy identified in the CFIR-ERIC Matching Tool, list each strategy’s feasibility and importance rating from the Go-Zone plot • Select strategies from the list recommended by the CFIR-ERIC tool considering each strategy’s feasibility and importance rating in the Go-Zone plot, use among other ePSMs reported in our scoping review, scope and target outcome, and applicability to the clinical contexts for the REACH system • Present implementation plan and obtain feedback from knowledge users
4. Produce implementation protocols and materials	Recommendations for specifying and reporting implementation strategies	• Specify how each strategy will be operationalized for REACH including the actors, actions, action targets, temporality, dose, and target outcomes

*CFIR* Consolidated Framework for Implementation Research, *ERIC* Expert Recommendations for Implementing Change, *ePSM* electronic Prospective Surveillance Model

### Steps 1 and 2: conduct an implementation needs assessment and identify implementation outcomes, performance objectives, determinants, and change objectives.

The implementation data previously collected from our scoping review and qualitative study (i.e., CFIR barriers and facilitators, potential actors and actions, and relevant outcomes) were used to complete Steps 1 and 2. To ensure selected implementation strategies were well suited to help overcome potential barriers, determinants to implementation were first assessed. Given that both our scoping review [27] and qualitative study [28] had developed separate lists of barriers and facilitators categorized by the CFIR, we aimed to integrate the findings from both studies to develop one list of CFIR constructs and their respective domains. This integration was performed two members of the team (CL and MT). The descriptions of the CFIR barriers and facilitators identified in the scoping review were used to confirm or complement the CFIR constructs identified in the qualitative study. However, some ePSM systems included in the scoping review differed significantly from our REACH system. Therefore, for each CFIR construct either identified across both studies, or only in our scoping review, specific factors highlighted in the descriptions from the scoping review were examined to see if they were applicable to the implementation of REACH. Barriers irrelevant to the REACH system were not included in the final list of determinants. Any disagreements were resolved through discussion between both team members and with assistance of a third team member (SNS). Additionally, barriers highlighted in the scoping review that were already being addressed by the REACH system were described as facilitators within the final list of determinants. The list of CFIR constructs was subsequently presented to and reviewed by the PFAC, Steering Committee, and advisors with expertise in implementing digital health interventions.

We used findings from our qualitative study to develop an initial list of relevant implementation actions and actors. During the qualitative interviews, participants were asked about the optimal time to invite patients to register to REACH, which clinic roles would be responsible for introducing and explaining the system to patients, and the type of information and materials about REACH that should be developed for patients and clinic staff. Implementation outcomes were identified using findings from our scoping review, which provided a list of ePSM systems that used each outcome from the implementation outcomes taxonomy [32]. The scoping review also offered possible measures for each outcome when evaluating the implementation of an ePSM. We aimed to use each outcome from the taxonomy to ensure a comprehensive evaluation of the implementation of REACH.

### Step 3: select theoretical change methods and design implementation strategies.

#### Step 3.1: develop a preliminary list of implementation strategies

Next, a preliminary list of potentially feasible and important implementation strategies that may address barriers identified in Step 1 was compiled, following definitions found in the Expert Recommendations for Implementing Change (ERIC) taxonomy [30, 31]. The preliminary list was developed using the CFIR-ERIC Implementation Strategy Matching Tool [34] and the Go-zone plot developed by Waltz et al. [31]. The CFIR-ERIC Matching Tool provides a list of recommended ERIC strategies to address specific CFIR barriers. The tool’s recommendations are based on the aggregated responses of implementation researchers and practitioners who were asked to rank up to seven ERIC strategies for each CFIR construct. The tool allows users to select the identified CFIR barriers and subsequently generates a list of strategies ordered by their cumulative level of endorsement. Level 1 endorsement refers to strategies with at least 50% of experts ranking the strategy as one of their top seven strategies for that barrier. Level 2 endorsement refers to strategies with 20–50% of endorsement. As such, each CFIR construct identified in Step 1 was entered into the CFIR-ERIC tool, and the implementation strategies recommended by the tool were organized by their thematic clusters. The CFIR constructs that were exclusively described as facilitators were not entered. The Go-zone plot was used to identify strategies suggested to be feasible and important. The Go-zone plot provides each ERIC strategy with a feasibility and importance score. The scores are based on the aggregated responses of implementation science and clinical experts who were asked to rate each strategy for its feasibility and importance. Each feasibility and importance rating from the Go-zone plot was mapped to each corresponding ERIC strategy recommended in the CFIR-ERIC Matching Tool.

#### Step 3.2: selection of implementation strategies for REACH

The selection of implementation strategies was led by a team member with training in implementation science (CL) with guidance from other team members with expertise in this field (JMJ and SNS). Several factors were considered for each strategy identified in Step 3.1 to help select implementation strategies for REACH. For each strategy identified from the CFIR-ERIC Matching Tool [31], two team members (CL and MT) compared the feasibility and importance ratings from the Go-zone plot to the strategy’s use among other ePSMs reported in our scoping review. Discrepancies between the feasibility and importance ratings from the Go-zone plot and the use of the strategy among other ePSMs were examined. For instance, ERIC strategies that were rated low in feasibility and/or importance in the Go-zone plot, but frequently used by other ePSMs were examined for their potential for impact and feasibility to the context of the setting implementing REACH (e.g., how the strategy would be employed, resources needed and available to deliver the strategy, and the individual responsible for carrying out each strategy). The selection of strategies was an iterative process and team members met regularly to reach consensus on the list of strategies for REACH. This iterative process also involved presenting the proposed implementation plan to the PFAC, Steering Committee, and advisors with expertise in implementing digital health interventions to obtain their feedback on the strategies selected, methods of administering the strategies, and important implementation barriers they might address.

Building upon these steps, further consideration was given to ensuring that the final list of strategies was diverse in scope (targeted different levels, including individuals and organizations, as well as different outcomes) and applicable to the active implementation phase for REACH. As such, we aimed to identify strategies that were similar in scope and consolidate the list of potential strategies to use for REACH. Strategies deemed relevant for or already used during the pre-implementation planning phase of this project were not selected.

Following the selection of strategies, the team met with the project’s PFAC and conducted a series of presentations across each centre with oncologists at disease site rounds and nurse and radiation therapy staff meetings to present and obtain feedback on the implementation plan. Finally, the implementation plan was presented and discussed with the Steering Committee. Following these presentations and considering feedback from the PFAC, Steering Committee, and advisors with expertise in implementing digital health interventions, team members with expertise in implementation science finalized the implementation plan.

### Step 4: produce implementation protocols and materials.

As described in Step 3.2, the selection of strategies encompassed considerations for how the strategy would be employed in the implementation settings (actors and actions) and the resources required to administer the strategy. Following these discussions, feedback, and the final selection of strategies, each strategy was specified according to the recommendations for specifying and reporting implementation strategies to advance our understanding of how, why, when, and where certain strategies work for a clinical innovation [35]. These include specifying the 1) actor (individual(s) enacting the strategy), 2) action (the specific steps or processes that need to be enacted), 3) action targets (the unit of analysis for measuring implementation outcomes such as clinicians or centres), 4) temporality (the timepoint and order the strategy is used), 5) dose (the frequency and duration of the strategy), 6) implementation outcomes addressed, and 7) justification (empirical, theoretical, or pragmatic reasons for selecting the strategy). The initial specification was completed two team members (CL and MT) and reviewed by the project’s Steering Committee.

## Results

### Steps 1 and 2: conduct an implementation needs assessment and identify implementation outcomes, performance objectives, determinants, and change objectives.

The synthesized list of barriers and facilitators across the scoping review and qualitative study is displayed in Table 2. Of the 39 CFIR constructs, 22 were identified as relevant determinants to the implementation of REACH; 15 of these were identified through both the scoping review and qualitative study, 4 from the scoping review alone and 3 from the qualitative study. Notably, while the specific descriptions of constructs that were either identified across both studies or only in our scoping review needed to be refined to ensure they were applicable to REACH, none of these constructs were excluded from the final list. The most common constructs were located within the intervention characteristics and inner setting domains. Few constructs identified were within the characteristics of individuals or process domains.

**Table 2:** Synthesized list of CFIR barriers and facilitators

CFIR Construct	Qualitative Study	Scoping Review	Barrier or Facilitator	Description
**Intervention Characteristics**				
Adaptability	✓	✓	Barrier	• Difficult to tailor the system to the variability of patients, including the treatment options and durations, languages spoken, and comfort with technology
			Facilitator	• The flexibility of when patients can register to the system and the ability to access the system on any electronic device • The ability to tailor the symptom screening questions to the cancer type and treatment status of the patient • The ability to offer resources through different modes of delivery (e.g., reading material, videos, online and in-person programs)
Relative Advantage	✓	✓	Barrier	• Concerns about the possible redundancy of the system with questions and recommendations from health care providers to manage cancer-related impairments • Concerns about replacing or decreasing the personal contact and discussions with health care providers
			Facilitator	• Patients receive an immediate recommendation on the system to manage their impairments • Potential improvements in processes for patients to access cancer rehabilitation resources • Provides patients with a centralized place to access trustworthy information
Complexity	✓	✓	Barrier	• Concerns about patients’ ability to use the system independently (navigating the system or difficulty understanding the screening questions) • Concerns about how to manage technical challenges or questions patients may have • Challenges managing concerning symptoms remotely
			Facilitator	• Ensure patients are aware of the remote nature of the system and that scores are not monitored by a health care provider
Design Quality and Packaging	✓	✓	Facilitator	• The ability for patients to view how their scores compare with their last assessment • The ability to save the resources recommended to view at a later time • Ensure the resources recommended are up to date
Evidence Strength and Quality	✓	✕	Barrier	• Skepticism of the system’s benefits on clinical and health service outcomes • Skepticism of the validity of the screening questions patients are asked to complete for each symptom
Cost	✕	✓	Facilitator	• There is no cost associated with registering and using the system • Recommended resources on the system are free for patients to access • Funding for creation, updating, and maintenance has been obtained
**Outer Setting**				
Patient Needs and Resources	✓	✓	Facilitator	• The potential to fill gaps in care by providing patients with resources and supports to manage their impairments • Provides a sense of empowerment and control • Provides a sense of reassurance and reduced uncertainty about symptoms
Cosmopolitanism	✓	✓	Barrier	• Concerns about the limited number of rehabilitation services and their capacities to respond to impairments identified by the system
			Facilitator	• The potential to build local connections between the cancer centre and community programs and services
External Policy and Incentives	✓	✕	Facilitator	• Ensuring institutional departments and teams such as privacy, security, and legal are engaged and that the system meets all necessary standards
**Inner Setting**				
Structural Characteristics	✓	✕	Barrier	• Centres where disease site clinics (e.g., breast, lymphoma) or disciplines (e.g., surgical oncology and medical oncology) are dispersed or located in different settings, may require more time or effort to implement the system due to having different workflows to consider and more staff to engage • Patients may be receiving treatment (e.g., surgery) at additional sites outside the cancer centre and therefore may have fewer opportunities to learn about the system
Implementation Climate (sub-constructs compatibility and relative priority)	✓	✓	Barrier	• Concerns about the potential overlap with existing or new electronic patient-reported outcomes systems used in the setting • Other initiatives and projects may be prioritized over the system by the setting and delay or hinder the implementation of the system • Potential previous unsuccessful experiences with implementation initiatives
			Facilitator	• Integrating the approach of registering patients on the system into processes used to communicate with patients and to provide patients with educational materials
Readiness for Implementation (sub-construct available resources)	✓	✓	Barrier	• Limited time for staff to introduce the system to patients during clinic visits • Concerns about the ability for the setting to respond to an increase in patient calls or visits as a result of the system
Networks & Communications	✕	✓	Facilitator	• Agreement about the division of roles and responsibilities among the implementation, clinical, and development teams
**Individual Characteristics**				
Knowledge and Beliefs	✓	✓	Barrier	• Belief that offering patients the opportunity to register and use the system is within staff’s scope of responsibilities
			Facilitator	• Ensuring patients and staff are familiar with the characteristics of the system and how the system is different from other electronic systems used by patients in the setting
Other Personal Attributes	✕	✓	Barrier	• No prior experience, comfort, or access to technology and internet • Patient is too ill or forgets to complete the assessments on the system
**Process**				
Engaging (sub-constructs opinion leaders and key stakeholders)	✓	✓	Barrier	• The ability for patients to self-register may lead to some patients being unaware of various features of the system
			Facilitator	• Ensuring clinic leadership and management are engaged and provide approval to implement the system in the setting • The ability to receive feedback on how the system can be integrated into the clinic workflow and a patient’s cancer pathway • Ensuring patients and staff are provided with engaging educational material to improve the adoption and uptake of the system • Ensuring patients are provided with reminders on the system to complete their symptom reporting • Availability of support for technical issues
Reflecting & evaluating	✕	✓	Facilitator	• Use of a flexible and iterative approach to implementation • Use of data and regular meetings with stakeholders to track and monitor implementation

*CFIR* Consolidated Framework for Implementation Research

For constructs identified in the scoping review, there were notable barriers that were deemed irrelevant to the implementation of REACH. For example, other ePSMs required patients to complete assessments in-clinic to inform the visit and provided HCPs with a summary report with recommended clinical actions and referrals. While the CFIR constructs related to these barriers remained in the final list, specific barriers related to these actions such as the high volume of alerts to HCPs (complexity) and challenges integrating the assessment reports into the electronic medical record (implementation climate) were not included in the description of their respective CFIR constructs.

The three CFIR constructs only identified in the qualitative study included ensuring HCPs believe in the benefits of the system (evidence strength and quality), engaging privacy, security, and legal teams at the institutions (external policies and incentives) and ensuring there are sufficient resources in place to implement REACH within centres where clinics are located in different spaces and function independently (structural characteristics).

The four constructs only identified in the scoping review that required further review for their applicability to REACH included cost, networks and communications, other personal attributes, and reflecting and evaluating. Each of these constructs remained on the list of barriers and facilitators considered in the REACH implementation plan. For instance, intervention development and delivery costs were key barriers in the scoping review. REACH is a free tool for patients, and funding for creation, updating, and maintenance has been obtained; however, cost should be considered for long-term sustainability.

Relevant implementation actions and actors for REACH are displayed in Table 3. Examples of actions identified to support the implementation of REACH included offering and explaining REACH to eligible patients at appropriate time points throughout the cancer pathway. Potential actors identified for this action were HCPs who have roles in patient education, such as ambulatory clinic nurses and radiation therapists, as well as information technology teams who may be able to develop automated and electronic processes to promote REACH to patients. Further, clinic leadership including physician site leads, nurse managers, and radiation therapy managers were identified as important actors who could assist with communicating the need for and benefit of REACH, and tailor the implementation to the clinical setting. Lastly, managing technical issues encountered by patients using the system was identified as a critical action to ensure implementation success, and potential actors included information technology teams and members of the REACH team such as the developers.

**Table 3:** Identified implementation actors and actions for REACH

Actor	Action
Clinic leadership	
Program and Clinical Directors	• Approve the addition of clinic actions and roles related to the implementation effort
Managers (nursing, radiation therapy, chemotherapy unit)	• Approve the addition of clinic actions and roles related to the implementation effort • Advocate, support, and communicate the need and benefits of REACH to clinic staff • Organize and coordinate education sessions related to REACH for clinic staff • Provide ongoing feedback on local implementation challenges and solutions
Physician site leads (medical oncology, radiation oncology, surgical oncology, hematology oncology)	
Health Care Providers	
Nurses (clinical nurse coordinators, nurse navigators, specialized oncology nurse, infusion nurse, etc.)	• Introduce the REACH system to eligible patients and provide them with information on how to register
Radiation therapists	
Oncologists	• For patients recommended by REACH to schedule a visit with their oncologist for further assessment, review the list of suggested referrals to community and hospital services on the print-out provided to patients when determining the most suitable course of action
Information Technology Teams	• Integrate information related to REACH into existing electronic systems and communication channels used by patients and/or providers in the centre
REACH System Developers	• Develop the code required to generate the data needed to evaluate REACH • Manage technical issues related to REACH reported by patients
REACH Implementation Team	• Structure and manage the data generated by the REACH system • Develop educational material about REACH for patients and clinic staff • Manage general inquiries from patients about REACH

Critical implementation outcomes identified included reach, feasibility, fidelity, acceptability, appropriateness, cost, and sustainability. Reach (absolute numbers and demographic and clinical characteristics), feasibility (e.g., length of time to complete assessments, technical issues experienced) and fidelity (completion of assessments and use of resources recommended by the system) collected passively through the REACH system will be important outcomes to understand who is using REACH and how they are using it. Patient user feedback, through surveys, will be used to further understand feasibility and acceptability of the system, and perceived appropriateness of the recommended resources. The costs related to any system adaptations and maintenance and will be reported. Lastly, sustainability will be considered by understanding the impact on available clinic processes and resources. As REACH may direct patients to rehabilitation services within the cancer centre, monitoring service usage overall, and appropriate service usage will provide insight into the impact of REACH on clinic resources and service wait times.

### Step 3: select theoretical change methods and design implementation strategies

#### Step 3.1: develop a preliminary list of implementation strategies

The preliminary list of 63 strategies with Level 1 (*n* = 50, 68%) or 2 (*n* = 13, 18%) endorsement from the CFIR-ERIC tool are displayed in Table 4, alongside the feasibility and importance ranking from the Go-zone plot. Notably, these endorsement levels were derived from the established criteria in the CFIR-ERIC Matching Tool and were not newly calculated for this study. Most of the identified strategies were related to developing stakeholder interrelationships (*n* = 16, 25%), training and educating stakeholders (*n* = 11, 17%), and using evaluative and iterative strategies (*n* = 10, 16%). Across the 63 strategies, 31 (49%) were ranked highly feasible and important within the Go-zone plot, nine (14%) were ranked highly feasible, but not important, eight (13%) were important, but not feasible, and 15 (24%) were ranked low in feasibility and importance.

**Table 4:** Results of CFIR-ERIC matching tool and feasibility and importance ranking from the literature

ERIC Cluster and Strategy	Cumulative Endorsement Percentage	Level 1 and 2 Endorsed CFIR Barriers	Go-Zone Quadrant
**Develop Stakeholder Interrelationships**			
Identify and prepare champions	418%	Evidence strength & quality Relative advantage Adaptability Complexity Structural characteristics Compatibility Knowledge & beliefs Opinion leaders Key stakeholders Patients/consumers	I
Build a coalition	286%	Cosmopolitanism Structural characteristics Compatibility Opinion leaders Key stakeholders	I
Conduct local consensus discussions	352%	Evidence strength & quality Relative advantage Adaptability Patient needs & resources Compatibility Relative priority Opinion leaders Key stakeholders	I
Inform local opinion leaders	259%	Evidence strength & quality Relative advantage Knowledge & beliefs Opinion leaders Key stakeholders	I
Capture and share local knowledge	264%	Evidence strength & quality Adaptability Complexity Cosmopolitanism Structural characteristics Available resources Knowledge & beliefs	I
Identify early adopters	215%	Evidence strength & quality Adaptability Complexity Structural characteristics Knowledge & beliefs Opinion leaders	I
Use advisory boards and workgroups	182%	Patient needs & resources Cosmopolitanism Key stakeholders Patients/consumers	I
Promote network weaving	148%	Cosmopolitanism Structural characteristics	III
Visit other sites	145%	Relative advantage Cosmopolitanism	II
Involve executive boards	122%	Cosmopolitanism Key stakeholders	II
Develop academic partnerships	116%	Evidence strength & quality Cosmopolitanism	II
Recruit, designate, and train for leadership	109%	Opinion leaders	IV
Model and simulate change	108%	Complexity	II
Organize clinician implementation team meetings	96%	Complexity	I
Use an implementation advisor	95%	None	I
Obtain formal commitments	75%	None	IV
**Use Evaluative and Iterative Strategies**			
Assess for readiness and identify barriers and facilitators	351%	Relative advantage Adaptability Complexity Patient needs & resources Structural characteristics Compatibility Relative priority Knowledge & beliefs Key stakeholders	I
Conduct local needs assessment	293%	Relative advantage Adaptability Patient needs & resources Compatibility Relative priority Knowledge & beliefs Key stakeholders	I
Conduct cyclical small tests of change	210%	Relative advantage Adaptability Complexity Structural characteristics Compatibility	I
Obtain and use patients/consumers and family feedback	187%	Patient needs & resources Patients/consumers	I
Develop a formal implementation blueprint	124%	Complexity	I
Stage implementation scale up	111%	Complexity Available resources	I
Purposefully re-examine the implementation	101%	Compatibility	I
Audit and provide feedback	86%	None	I
Develop and implement tools for quality monitoring	53%	None	I
Develop and organize quality monitoring systems	45%	None	I
**Train and Educate Stakeholders**			
Conduct educational meetings	263%	Evidence strength & quality Relative advantage Knowledge & beliefs Opinion leaders Key stakeholders	I
Create a learning collaborative	219%	Adaptability Complexity Cosmopolitanism Key stakeholders	II
Conduct educational outreach visits	169%	Evidence strength & quality Cosmopolitanism Knowledge & beliefs	II
Develop educational materials	177%	Evidence strength & quality Knowledge & beliefs Patients/consumers	I
Distribute educational materials	110%	Evidence strength & quality	I
Provide ongoing consultation	92%	Complexity	I
Conduct ongoing training	75%	Complexity	I
Work with educational institutions	59%	None	II
Use train-the-trainer strategies	56%	None	I
Shadow other experts	44%	None	II
Make training dynamic	40%	None	I
**Adapt and Tailor to Context**			
Promote adaptability	285%	Relative advantage Adaptability Complexity Structural characteristics Compatibility	I
Tailor strategies	223%	Adaptability Complexity Compatibility	I
Use data experts	46%	None	III
**Provide Interactive Assistance**			
Facilitation	173%	Adaptability Complexity Compatibility Knowledge & beliefs	I
Provide local technical assistance	72%	None	IV
Centralize technical assistance	36%	None	III
**Support Clinicians**			
Develop resource sharing agreements	80%	Cosmopolitanism Available resources	III
Facilitate relay of clinical data to providers	65%	None	I
Revise professional roles	48%	None	III
Create new clinical teams	40%	None	III
**Engage Consumers**			
Involve patients/consumers and family members	236%	Patient needs & resources Opinion leaders Patients/consumers	I
Prepare patients/consumers to be active participants	118%	Patient needs & resources Patients/consumers	IV
Increase demand	115%	Relative advantage Relative priority Available resources	II
Intervene with patients/consumers to enhance uptake & adherence	106%	Patient needs & resources Patients/consumers	IV
Use mass media	90%	Patients/consumers	III
**Use Financial Strategies**			
Alter incentive/allowance structures	179%	Relative advantage Relative priority	III
Access new funding	134%	Available resources	IV
Fund and contract for clinical innovation	103%	Available resources	IV
Alter patient/consumer fees	44%	Available resources Patients/consumers	III
Place innovation on fee for service lists/formularies	38%	None	IV
Make billing easier	32%	Available resources	III
**Change Infrastructure**			
Change physical structure and equipment	97%	Structural characteristics Available resources	III
Mandate change	65%	Relative priority	III
Create or change credentialing and/or licensure standards	41%	None	III
Change service sites	38%	None	III
Change record systems	29%	None	III

*CFIR* Consolidated Framework for Implementation Research, *ERIC* Expert Recommendations for Implementing Change; Cumulative Endorsement Percentage, collective percentages of all endorsement across all identified CFIR barriers; Go-zone quadrant I, high importance and feasibility; Go-zone quadrant II, low importance and high feasibility; Go-zone quadrant III, low importance and feasibility; Go-zone quadrant IV, high importance and low feasibility

#### Step 3.2: selection of implementation strategies for REACH

The final list of strategies and rationale for selection are displayed in Table 5. The selection of these strategies involved careful consideration of various factors (e.g., feasibility, potential for impact, applicability to the clinical context, use among other ePSMs, and similarity with other potential strategies identified), as well as consideration of the feedback from various knowledge users, including feedback on addressing barriers within key domains and constructs (e.g., compatibility within the inner setting domain, complexity within the intervention characteristics domain, and engaging within the process domain). For example, clinic staff, members from the PFAC, and Steering Committee all highlighted the importance of ensuring that important outcomes such system usage and feasibility, and barriers to implementation were regularly monitored to address any potential system or implementation issues. The strategies ‘conduct cyclical tests of change’, and ‘purposefully re-examine the implementation’ would likely achieve this objective and both were similar in scope as they aim to monitor the success of the implementation effort and continuously refine the implementation plan. Both were identified as feasible and important, with a high cumulative endorsement rate via the CFIR-ERIC tool for addressing barriers related to compatibility within the inner setting. We chose to focus on ‘purposefully re-examine the implementation’ as it could be more broadly applied and tailored to include the use of cyclical small tests of change.

**Table 5:** Description and justification of implementation strategies selected

ERIC Cluster and Strategy	ERIC Description and Ancillary Material	Justification
**Use evaluative and iterative strategies**		
Purposefully re-examine the implementation	Monitor progress and adjust clinical practices and implementation strategies to continuously improve the quality of care. It is beneficial to use a concrete schedule for monitoring rather than ‘as needed.’	Data considered: • 101% endorsement via CFIR-ERIC tool • High in feasibility and importance (Quadrant I) • Low reported usage among other ePSM evaluations REACH project considerations: • Feasible and important to regularly monitor implementation success, barriers to implementation, and use of other implementation strategies • Similar to strategies such as ‘conduct cyclical test of change’ and ‘develop and organize quality monitoring systems’ (i.e., to monitor the success of the implementation effort and continuously refine the implementation plan), but can be broadly applied and tailored to include cyclical tests of change if necessary CFIR constructs targeted by strategy: • Inner Setting – Compatibility • Intervention Characteristics – Complexity, Adaptability • Process – Engaging Key Stakeholders and Patients
**Change Infrastructure**		
Change record systems	Change records systems to allow better assessment of implementation or clinical outcomes. This strategy involves changing the structure, content, function, or design of record system components such as electronic medical records to reflect the innovation	Data considered: • 29% endorsement via CFIR-ERIC tool • Low in feasibility and importance (Quadrant III) • High reported usage among other ePSM evaluations REACH project considerations: • While each centre has their own information technical teams, embedding the process to offer REACH to patients within existing electronic and automated systems used to distribute information to patients was considered important • For sites that require clinic staff to offer REACH to patients, integrating this task into their workflow was considered important. This includes documentation checklists either in the electronic medical record or another system used in the setting. It was thought that this would act as a reminder and encourage providers to complete this task Barriers targeted by strategy: • Inner Setting – Compatibility
**Train and Educate Stakeholders**		
Conduct educational meetings	Hold meetings targeted toward educating multiple stakeholder groups (i.e., providers, administrators, other organizational stakeholders, community members, patients/consumers, families) about the clinical innovation and/or its implementation	Data considered: • 263% endorsement via CFIR-ERIC tool • High in feasibility and importance (Quadrant I) • High reported usage among other ePSM evaluations REACH project considerations: • Feasible and important to educate health care providers and administrative staff on the purpose of REACH, differences with other systems, and how REACH fits within their clinical workflow • The REACH team can tailor the number of meetings across different sites based on need Barriers targeted by strategy: • Individual Characteristics – Knowledge and Beliefs • Intervention Characteristics – Relative Advantage
Distribute educational materials	Distribute educational materials (including guidelines, manuals, and toolkits) in person, by mail, and/or electronically	Data considered: • 110% endorsement via CFIR-ERIC tool • High in feasibility and importance (Quadrant I) • High reported usage among other ePSM evaluations REACH project considerations: • Feasible and important to effectively distribute educational materials to patients to ensure they can easily register and use the tool independently Barriers targeted by strategy: • Individual Characteristics – Knowledge and Beliefs
**Engage Consumers**		
Intervene with patients to enhance adherence and uptake	Develop strategies with patients to encourage and problem solve around adherence. This includes patient reminders and financial incentives. Feedback regarding patients’ understanding and use of the innovation is also important to collect	Data considered: • 106% endorsement via CFIR-ERIC tool • Low in feasibility and high in importance (Quadrant IV) • High reported usage among other ePSM evaluations REACH project considerations: • Given that REACH is a remote self-management tool with a low frequency of assessments, and that the use of the tool is not linked to a clinic visit, it is important to develop a system on REACH to remind and encourage patients to log in and complete their assessments when asked Barriers targeted by strategy: • Individual Characteristics – Knowledge and Beliefs • Process – Engaging Patients
**Provide interactive assistance**		
Centralize technical assistance	Develop and use a centralized system to deliver technical assistance focused on implementation issues. This could be the designation of a lead technical assistance organization (could also be responsible for training). The lead technical assistance entity can develop other mechanisms (e.g., call-in lines or websites) in order to share information on how to best implement the clinical innovation	Data considered: • 36% endorsement via CFIR-ERIC tool • Low in feasibility and importance (Quadrant III) • Low reported usage among other ePSM evaluations REACH project considerations: • Given that REACH is in its initial version and each centre has their own information technical teams, it was thought that centralizing technical assistance using personnel within the REACH team would provide a feasible method to monitor technical issues and coordinate with the REACH development team during the pilot phase, rather than developing a system for each site to deliver technical assistance on local implementation issues Barriers targeted by strategy: • Intervention Characteristics – Complexity • Individual Characteristics – Knowledge and Beliefs • Outer Setting – Patient Needs and Resources
**Develop stakeholder interrelationships**		
Use advisory boards and workgroups	Create and engage a formal group of multiple kinds of stakeholders to provide input and advice on implementation efforts and to elicit recommendations for improvements	Data considered: • 182% endorsement via CFIR-ERIC tool • High in feasibility and importance (Quadrant I) • Moderate usage among other ePSM evaluations REACH project considerations: • Involving various roles such as directors, managers, and clinicians will be critical to successfully conducting the strategy ‘purposefully reexamine implementation’ (see above) • Conducting meetings with these various roles at each site will provide the REACH team with a better understanding of current clinical workflows and any changes made, direction on when and how patients are invited to register to REACH, feedback on these processes, and approvals for implementing new processes Barriers targeted by strategy: • Inner Setting – Compatibility, Relative Priority • Process – Engaging Key Stakeholders and Patients • Outer Setting – Patient Needs and Resources
**Adapt and tailor to context**		
Tailor strategies	Tailor the implementation strategies to address barriers and leverage facilitators that were identified through earlier data collection	Data considered: • 223% endorsement via CFIR-ERIC tool • High in feasibility and importance (Quadrant I) • Low reported usage among other ePSM evaluations REACH project considerations: • REACH is being implemented within four centres. Structural differences will need to be considered when conducting several strategies such as how implementation teams are formed (organize implementation teams and team meetings), who educational meetings are targeted towards along with their frequency and duration (conduct educational meetings), and how educational materials are distributed to patients (distribute educational materials) Barriers targeted by strategy: • Inner Setting – Compatibility • Intervention Characteristics – Complexity, Adaptability

*ERIC* Expert Recommendations for Implementing Change, *ePSM* electronic prospective surveillance model, *CFIR* Consolidated Framework for Implementation Research

While ‘provide local technical assistance’ had a higher feasibility and importance ranking and endorsement rate than ‘centralize technical assistance’, the latter was selected due to fit with the local context. As REACH is in its initial version and each centre has their own information technology teams, it was thought that centralizing technical assistance using the REACH personnel and a dedicated REACH email account would provide a feasible method to monitor and respond to technical issues during the pilot phase. Furthermore, feedback from the PFAC helped refine how this strategy would be administered, including suggestions to include an automated response which indicates that a team representative will respond as soon as possible with a link to the About REACH section within the system, as well as a target timeframe that REACH personnel should respond to patient inquiries (i.e., 48 h). Similarly, the strategy ‘change record systems’ was selected despite having a 29% endorsement rate via the CFIR-ERIC Matching Tool and rated low in feasibility and importance via the Go-Zone plot. Through our scoping review, this strategy was identified as highly utilized among other ePSMs by integrating the system into the centre’s electronic medical record or other electronic systems. Further, feedback from clinic staff, the project’s Steering Committee, and advisors with expertise in implementing digital health interventions highlighted the importance of addressing barriers related to the inner setting, specifically the compatibility of REACH with existing workflows and that leveraging existing electronic and automated processes to deliver information to patients and clinic staff may support patient registration onto the system.

There were 5 strategies that were used in the pre-implementation planning phase for REACH and therefore marked off as completed. These included ‘assess readiness and identify barriers and facilitators’, ‘inform local opinion leaders’, ‘visit other sites’, ‘involve patients and family members’, and ‘use data experts’. However, some of the components within these strategies were embedded within selected strategies for the implementation phase. For instance, the strategies ‘assess for readiness and identify barriers and facilitators’ and ‘involve patients and family members’ were previously utilized to facilitate the development of REACH. The assessment of barriers will continue throughout the implementation of REACH through the strategy ‘purposefully re-examine the implementation’ and the involvement of patients will continue through the strategy ‘use advisory boards and workgroups’.

Lastly, while several strategies within the cluster 'utilize financial strategies’ had a high endorsement rate and were identified as important (e.g., ‘access new funding’ and ‘fund and contract for the clinical innovation’), these strategies were not selected for the initial implementation of REACH. Rather, these strategies will form part of the formal sustainability discussions and planning conducted throughout this initial implementation phase.

### Step 4: produce implementation protocols and materials

Table 6 displays how each strategy was operationalized for REACH. Notably, the strategy ‘purposefully re-examine the implementation’ will provide a foundation for the approach to implementing REACH. Throughout the 16-month evaluation of the implementation of the system, we will routinely monitor key implementation outcomes, adjust the functionality of REACH if necessary, and modify the implementation strategies used to promote the system to improve uptake and user experience.

**Table 6:** Proposed operationalization of implementation strategies selected for REACH

Strategy	Actor	Action	Action Target	Temporality	Dose	Target Outcome
Purposefully re-examine the implementation	REACH team members	Monitor implementation outcomes using data collected from the REACH system, patient feedback, and feedback from clinic staff to inform changes to the implementation effort	Improved understanding of implementation success, barriers to implementation, and changes made throughout the implementation effort	Outcomes will be summarized every 4 months following the go-live date for each centre	Use of 4, 4-month plan-do-study-act cycles	Reach, acceptability, appropriateness, feasibility, fidelity, and sustainability
Tailor strategies	See actors for each specific strategy	Certain strategies will be tailored to address barriers and leverage facilitators specific to each site. This may include the action, action target, temporality, and dose. Tailoring is specified for each strategy where appropriate	See action targets for each specific strategy	See temporality for each specific strategy	See dose for each specific strategy	See outcomes for each specific strategy
Change record systems	Information technology teams within each site	Integrate information about REACH into other electronic systems used by the clinical site. Strategy will be tailored to each site’s availability of other systems	Patients eligible for REACH may be provided with information about the tool directly through a patient portal or other system Clinic staff responsible for introducing REACH to eligible patients may be provided with a prompt in the electronic medical record to document the completion of this task	Changes will be made for the launch date and additional changes will be made if necessary throughout the implementation phase	Once, followed by additional changes if needed	Reach, sustainability
Conduct educational meetings	REACH team member	Attend and present at oncology rounds, nursing rounds and other team meetings	Tailored to each site depending on how patients are being introduced to REACH. May include physicians, nurses, and/or radiation therapists Improved awareness and knowledge of REACH, including the symptoms screened and resources provided to patients	Presentations should be delivered within one month of launching REACH Additional meetings may be conducted	Tailored to each site Frequency: One, followed by additional meetings if needed Duration: 10–30 min (based on preferences and feasibility at each site) Format: In-person or virtual, synchronous or asynchronous (based on preferences and feasibility at each site)	Reach
Distribute educational materials	REACH team members and clinic leadership	Prepare pathways to distribute a handout about REACH to patients (tailored to each site depending on clinic preferences and available resources). This may include an online education prescription system, a patient portal, physical or electronic education packages, group virtual or in-person education classes, and displays at the clinic registration desks	Enable patients to register and use the tool independently	Pathways to distribute the REACH handout should be prepared within one week prior to the go-live date	Changes to the how, when and how often patients are provided the handout will be made if needed	Reach, sustainability
Intervene with patients to enhance adherence and uptake	REACH development team	REACH will provide automated reminders via email to patients to complete their assessment if not completed	Enable patients to log in and complete assessments to receive resources	REACH reminders will begin once the patient has registered continue for each assessment	Patients will receive up to 2 reminders for each incomplete assessment 2 days and 3 days after the initial prompt	Fidelity
Centralize technical assistance	REACH team members	The REACH system will provide an email to ask questions about the system and report technical issues for the purpose of creating a help section with frequently asked questions after the implementation pilot	Patients with or without an active account on REACH Improved understanding of common questions and technical issues with the system Improved understanding of resources needed to ensure sustainability of system following implementation pilot	The email account should be developed before the roll-out of the system and assessed throughout the implementation effort	Responses to patient inquiries will be answered within 48 h	Feasibility
Use advisory boards and workgroups	REACH team members	Feedback from clinic staff working in each implementation site will be solicited. Meetings will be held with staff to provide an update on the implementation effort and aim to provide staff and REACH team members with protected time to reflect on the implementation effort, share lessons learned through collaborative meetings, and discuss practical issues, barriers, and possible solutions. Meetings will also involve mapping work processes and planning changes to how patients are introduced to REACH	Clinic staff (e.g., directors, managers, site leads) Improved understanding of current clinical workflows and any changes made, obtain feedback and approvals for how patients are invited to register to REACH Strategy may be tailored to involve multiple meetings with individuals depending on the availability of individuals and specific barriers and solutions to discuss	The first meeting should be held in the first month of the initial roll-out	The frequency, number, and duration of meetings will be tailored to each site. At least one meeting will be held during each 4-month plan-do-study-act cycle	Reach, sustainability

## Discussion

This article describes the process of applying the step, ‘select and tailor strategies’, within the KTA cycle using an IM approach to develop an implementation strategy for an ePSM, called REACH. This process led to a multifaceted implementation strategy that targets multiple levels of implementation, including individual- and organizational-level strategies. These include training and educating patients and clinic staff about REACH, using of automated system reminders to ensure REACH is used as intended, regularly engaging clinic leadership to discuss barriers and possible solutions to implementation, providing patients with technical support, leveraging or modifying existing electronic systems used in each setting to facilitate the delivery of information about REACH to patients, and monitoring the quality of implementation to inform changes to the system and implementation plan.

IM provided a generalizable and pragmatic approach to selecting implementation strategies for REACH. This approach encouraged the meaningful engagement of stakeholders and the use of relevant implementation science frameworks. IM has been used for numerous community and clinical contexts and has been applied using a variety of methods [36]. Our approach to applying IM encompassed notable considerations to the context of implementing the REACH system. The identification of barriers, actions, and actors was conducted during the process of developing the REACH system. As such, we had sufficient time to conduct a scoping review and qualitative study and identify a comprehensive list of potential barriers to implementation guided by the CFIR. In fact, this preliminary work was conducted over a period of approximately 18–24 months, with the IM process taking an additional 6 months. However, the use of these methods may not be practical for settings implementing ePSMs that are constrained by timelines. These settings may benefit from incorporating rapid qualitative assessments, which have shown promise in balancing rigour and efficiency and may facilitate the use of real-time data to inform implementation decisions [37]. Further, the use of online surveys to obtain insights from stakeholders into barriers and facilitators categorized by implementation science determinant frameworks such as the CIFR and the Theoretical Domains Framework [38] is another approach that can also be combined with qualitative interviews. This approach has led to the development of a tailored implementation plan for systems using ePROs in clinical settings [39].

To leverage identified barriers and inform the selection of implementation strategies, we utilized the CFIR-ERIC Matching Tool [34]. Our approach was slightly different to other methods of identifying implementation strategies for self-management interventions in cancer care. For instance, Howell et al. [40] categorized identified barriers and enablers from qualitative interviews to the CFIR prior to identifying strategies using the CFIR-ERIC Matching Tool. However, given the high volume of strategies that were recommended for the REACH system, we took additional steps to consolidate the list of strategies by comparing their aims, feasibility and importance, and their use among other ePSM systems identified by our scoping review. This approach is similar to the one by Verweij et al. [41], which included prioritizing barriers based on three levels prior to using the CFIR-ERIC Matching Tool, and re-engaging key stakeholders to refine the implementation plan. Taking these additional steps reflects the limitations of the CFIR-ERIC Matching Tool and aligns with recommendations that suggest using the tool as a starting point for planning and organizing discussions to tailor the implementation to the clinical context [34]. Our approach to considering additional contextual factors reflects the approach by Kennedy et al. [42] who developed a tailored multifaceted strategy for an exercise oncology program. While Kennedy et al. [42] did not use the CFIR-ERIC Matching Tool, the final list of ERIC strategies was developed based on feedback from stakeholders that considered each strategy’s ability to address identified barriers, its feasibility, and impact on implementation success.

The use of multifaceted and tailored strategies that target multiple relevant implementation determinants has been suggested to be more effective than discrete strategies [20, 43]. This may be particularly important given that evidence-based interventions, clinical settings, and populations are highly heterogenous. While the evidence for the effectiveness of implementation strategies for integrating patient-reported outcome systems into cancer care is limited, the strategies selected for REACH reflect many of the actions that are recommended for these systems and perceived as important to support implementation. For instance, strategies recommended include those that seek to assess readiness and current work processes, adapt and tailor the implementation to the clinical context, engage clinical staff (e.g., training on the system, and providing feedback to clinics on the use of the system among patients and changes in symptom burden), and provide technical support [16, 32]. A pragmatic evaluation of the implementation of REACH is underway guided by the implementation outcomes framework and the Longitudinal Implementation Strategy Tracking System (LISTS) [44]. This evaluation will help identify critical strategies required for implementation success, as well as capture planned or unplanned adaptations to the implementation strategies (e.g., actions, dose, frequency), and the discontinuation or addition of strategies.

While our approach to selecting implementation strategies for REACH included the use of several implementation science frameworks, a comprehensive list of CFIR barriers generated through rigorous methods, and a broad group of stakeholders throughout this process, this approach also had several limitations. First, our approach primarily focused on mapping identified barriers to implementation strategies rather than leveraging facilitators to complement existing strengths at the individual and organizational level. There is a need for improved guidance on integrating the identification of facilitators into the process of selecting and tailoring implementation strategies, and this has been previously highlighted as a limitation of using the CFIR-ERIC Matching Tool [45]. Second, our engagement of stakeholders following the identification of barriers could have been strengthened. For instance, while the team presented the implementation plan to the project’s PFAC and clinic staff, there were important differences to the level of feedback and engagement between the two groups. The feedback from the PFAC was more in-depth likely because they had already met with the study team several times prior to this meeting, were very familiar with the REACH system, and the meeting provided protected and sufficient time (i.e., 1–2 h) to reflect on the implementation plan and provide meaningful feedback. Alternatively, the presentations with clinic staff were brief (i.e., 10–15 min) and clinic staff were less familiar with REACH. As such, feedback primarily related to the features and content of the system and the process for identifying and managing patients who report high scores on their assessments. Further, while meetings with clinic leadership provided important feedback about the implementation plan, our approach could have been strengthened by providing leadership with time outside of these meetings to reflect on the various components of implementation plan. For instance, Knapp et al. [46] incorporated a modified Delphi approach during the development of an implementation strategy, where members of the advisory panel, including clinicians, rated each proposed strategy’s perceived effectiveness, feasibility, and importance for their setting. This approach would have likely improved the quality and depth of the feedback from clinic leadership and strengthened the discussions of each strategy’s feasibility and importance for REACH.

## Conclusions

The implementation of ePSMs within the delivery of cancer care has the potential to improve the long-term function and quality of life of people living with and beyond cancer through the systematic identification and management of cancer-related impairments. However, the implementation of ePSMs is challenged by a limited understanding of optimal approaches to selecting appropriate implementation strategies for these systems. This article describes a generalizable and pragmatic approach for developing a tailored multifaceted implementation strategy for an ePSM for cancer rehabilitation, called REACH. The approach used for this project, which followed IM methodology and applied the CFIR and ERIC taxonomy, may provide guidance to researchers, clinicians, clinic leadership, and other stakeholders interested in implementing these systems into their oncology practices.

## Supplementary Information

Additional file 1: Standards for Reporting Implementation Studies Checklist

## Data Availability

The dataset used and analysed during the current study are available from the corresponding author on reasonable request.

## Abbreviations

PSM: Prospective Surveillance Model
ePSM: Electronic Prospective Surveillance Model
ePROs: Electronic patient reported outcomes
KTA: Knowledge to Action
IM: Implementation Mapping
ERIC: Expert Recommendations for Implementing Change
CFIR: Consolidated Framework for Implementation Research
LISTS: Longitudinal Implementation Strategy Tracking System
